# Clinicopathological Profile of Paraprotein-Associated Kidney Disease in Monoclonal Gammopathies: An Observational Study

**DOI:** 10.7759/cureus.32929

**Published:** 2022-12-25

**Authors:** Divya Parepalli, Bheemanathi Hanuman Srinivas, Debdatta Basu, Priyamvada PS, Biswajith Dubashi

**Affiliations:** 1 Pathology, Jawaharlal Institute of Postgraduate Medical Education and Research, Puducherry, IND; 2 Nephrology, Jawaharlal Institute of Postgraduate Medical Education and Research, Puducherry, IND; 3 Medical Oncology, Jawaharlal Institute of Postgraduate Medical Education and Research, Puducherry, IND

**Keywords:** mgrs, monoclonal gammopathy of renal significance, monoclonal immunoglobulin deposition disease, renal amyloidosis, light chain cast nephropathy, paraprotein associated kidney disease

## Abstract

Background

Renal involvement in monoclonal gammopathies presents with different clinico-morphological patterns and can manifest at the onset or the late phase of hematological disease, or after chemotherapy. The spectrum is ever-expanding with advancements in diagnostic methods. Renal biopsy is needed for accurate diagnosis, as each of these patterns carries therapeutic and prognostic implications.

Methods

A total of 41 cases of monoclonal gammopathies were included in the study. Clinical, biochemical, and hematological details were obtained, and pathological variables were observed. Patients were followed till the maximum possible period, and treatment history and follow-up creatinine details were collected.

Results

The spectrum of renal biopsy lesions observed included light chain cast nephropathy (LCCN) n=19, amyloidosis n=11, monoclonal immunoglobulin deposition disease (MIDD) n=6, and proliferative glomerulonephritis with monoclonal immunoglobulin deposition (PGNMID) n=5; 10 of these cases can be categorized as monoclonal gammopathy of renal significance (MGRS). Acute kidney injury (AKI) (41%) is the predominant clinical presentation in general whereas the majority of amyloidosis cases presented with nephrotic and sub-nephrotic proteinuria. LCCN cases had high serum creatinine and calcium, positivity for M-spike, as well as a high FLC ratio, compared to the other types. Around 100% of LCCN and MIDD patients had myeloma and 100% of PGNMID cases had normal marrow.

Conclusion

More than three-fourths of patients were diagnosed with monoclonal gammopathies with biochemical and hematological workups after an initial kidney biopsy. The clinicopathological profile of these patients had a broad spectrum but there were still some consistent findings within the different types. A subgroup of patients (MGRS) had undetectable serum paraproteins but had monoclonal immunoglobulin deposition in the kidney.

## Introduction

According to Santostefano et al., “monoclonal gammopathies (MG) include various clinicopathological entities characterized by B-cells or lymphoplasmacytic cell synthesis with tissue deposition of whole monoclonal immunoglobulin (Ig) or Ig subunits, constituting light chains (LC) or heavy chains (HC) or both (HLC)” [1]. These patients can have specific or non-specific clinical presentations with varied findings which can mimic many other diseases. Renal involvement presents with different clinico-morphological patterns, which can occur at the onset or late phase of hematological disease or after chemotherapy [1,2]. Sometimes renal involvement might be the sole finding in the patient with an undetectable clone in blood and bone marrow.

MG can involve either glomerulus, tubules, interstitium, and blood vessels alone or in combination. A morphologic diagnosis is seldom possible on clinical grounds. A renal biopsy helps in the accurate diagnosis of the pattern of injury, clonality, and ultra-structural details. It also aids in differentiating renal lesions due to plasma cell dyscrasias from its mimickers early in the course, as the etiology carries therapeutic and prognostic implications if the plasma cell disorder is identified early even before the hematological manifestations [2].

The typical renal lesions in MG include light chain cast nephropathy (LCCN), amyloidosis, and monoclonal immunoglobulin deposition disease (MIDD) [3]. However, the spectrum is expanding with advancements in diagnostic techniques [4-6]. Renal involvement as observed in MGRS even with low plasma cell burden may warrant treatment without which the survival of the organ is affected, leading to end-stage renal disease [7]. The recurrence of the disease in the graft is also earlier when compared with other lesions not associated with monoclonal gammopathies [8]. So knowledge of the clinical, pathological, and hematological spectrum of disease in these patients is vital for early detection, which helps in salvaging the kidney. Our objective is to observe the spectrum of paraprotein-associated disease in kidney biopsies and to correlate with their hematological and renal profile in patients with monoclonal gammopathies.

## Materials and methods

This study was approved by the Institute Ethics Committee for Human Studies at Jawaharlal Nehru Institute of Post Graduate Medical Education and Research (JIPMER), Puducherry, India. All diagnosed cases of monoclonal gammopathy with renal involvement (according to the Kidney Disease Improving Global Outcomes (KDIGO) guidelines) that were screened wherever the kidney biopsies were indicated from 2011 to 2018 were chosen for the study after including the cases where the study patient’s relevant clinical, hematological and biochemical parameters were obtained. Pathological parameters were examined for bone marrow aspirate as well as bone marrow and kidney biopsies, and patients were followed up for the maximum possible period.

Kidney biopsy light microscopy sections were stained by H&E, periodic-acid Schiff (PAS), Masson’s trichrome (MT), and periodic silver methenamine (PASM) and congo-red wherever needed. Immunohistochemical markers κ, λ, AA, CD138, CD38, CD20, CD56, and for immunofluorescence (IF) studies 3 micron cryostat sections were stained with fluorescein isothiocyanate conjugated antibodies IgG, IgM, IgA, κ, λ, C3, C1q. Electron microscopic (EM) examination of the tissue was not done in any of the cases due to the non-availability of EM at our institution.

Statistical analysis was done using Statistical Package for the Social Sciences (SPSS) software v.23 (IBM Corp., Armonk, NY). The categorical distribution is presented as frequency and percentages, and continuous variables are shown as mean with standard deviation. One-way ANOVA is used for finding a difference in means for parametric continuous variables among the kidney disease types. The chi-square test was used for finding an association of categorical variables like morphological findings, marrow plasma cell distribution pattern, marrow diagnosis, and urinary sediments in relation to kidney disease types. Differences were considered significant at a p-value of < 0.05.

## Results

The spectrum of paraprotein-associated kidney disease observed included LCCN in 19 patients (46%), amyloidosis (AA negative) in 11 patients (27%), MIDD in six patients (15%), and proliferative glomerulonephritis with monoclonal immunoglobulin deposits (PGNMID) in five patients (12%). Among the patients in the Bone marrow (BM) study, all the cases of LCCN and MIDD had myeloma, but only 5 (45%) patients of amyloidosis had myeloma and all cases of PGNMID had normal marrow findings.

 The overall mean age was 56 ± 9.6 years and 62% of these cases were in the age group of 41-60 (Table 1). The mean age of patients with PGNMID was 10 years more than patients with other lesions. Younger age at presentation with a mean age of 50 years was seen in the patients with MIDD. There was a male preponderance with a ratio of 3.1:1. On USG, there was normal mean kidney size in all disease types with relatively smaller sizes in MIDD.

**Table 1:** Demographic, serological and hematological variables n is the number of cases in which the details are available for the particular variable. LCCN: light chain cast nephropathy; MIDD: monoclonal immunoglobulin deposition disease; PGNMID: proliferative glomerulonephritis with monoclonal immunoglobulin deposition; SPEP: serum protein electrophoresis; IFE: immunofixation electrophoresis #chi-square test; *one way ANOVA. Among the 37 cases with available bone marrow biopsies, 28 are myeloma and 10 cases are monoclonal gammopathy of renal significance (MGRS).

Variable	Findings	All cases	LCCN	Amyloidosis	MIDD	PGNMID	P-value
No. of patients		41	19	11	6	5	
Age (mean ± SD)		56±9	55±8	56±10	50±9	64±9	0.1*
Male sex-number (%)		31(75)	15(78)	8(73)	4(67)	4(80)	0.92#
Serum calcium mg/dL (mean ± SD)		8.8±1.3	9.2±1.6	8.7±0.7	8.7±1.1	7.6±0.5	0.15*
(n-number of cases for which the serum calcium levels are available)		(n=37)	(n=17)	(n=10)	(n=6)	(n=4)	
SPEP-number (%)	Positive for M-spike	19(76)	11/11(100)	3(42.9)	5/5/(100)	-	0.002#
	Negative for M-spike	6(24)	-	5(57.1)	-	1	
(n- number of cases for which the SPEP details are available)		(n=25)	(n=11)	(n=8)	(n=5)	(n=1)	
IFE-number (%)							
κ		3(19)	2(33)	1(16.7)	-	NA	-
λ		2(13)	1(17)	-	1(25)		
IgG, κ		4(25)	2(33)	-	2(50)		
IgG, λ		5(31)	1(17)	4(66.7)	-		
IgM,λ		1(6)	-	1(16.7)	-		
IgA, λ		1(6)	-	-	1(25)		
(n-number of cases for which the IFE details are available)		(n=16)	(n=6)	(n=6)	(n=4)		
Hb mg/dl (mean ± SD)		8.8±1.9	8.2±1.8	10.1±1.9	8.18±1.4	8.9 ±1.4	0.03*
n (number of cases for which the Hb levels are available)		(n=41)	(n=19)	(n=11)	(n=6)	(n=5)	
Plasma cell % (mean ± SD)		36.3±25.4	53.2±19.2	19±14.6	37.2±27.4	5±3.4	0.00*
n (number of cases for which the plasma cell % are available)		(n=37)	(n=17)	(n=10)	(n=6)	(n=4)	
Plasma cell distribution pattern (BM biopsy)	Interstitial	27(73)	8(47)	10(100)	5(83)	4(100)	0.03#
	Nodular	3(8)	2(12)	-	1(17)		
	Diffuse	7(19)	7(41)	-	-		
(n-number of cases for which the plasma cell distribution pattern details are available)		(n=37)	(n=17)	(n=10)	(n=6)	(n=4)	

Among the kidney diseases, the most common clinical presentations were AKI in LCCN (58%), MIDD (50%) and PGNMID (40%), and nephrotic syndrome (46%) in amyloidosis (Table 2). In addition, many of the patients had extrarenal manifestations - organomegaly, cardiac and neural manifestations in 50% of the patients with amyloidosis. Cardiac manifestations were also observed in two of the LCCN and PGNMID and one of the MIDD cases. 

**Table 2:** Renal parameters #chi-square test; *one-way ANOVA. Semi-quantitative grading was used for tubular atrophy/interstitial fibrosis. LCCN: light chain cast nephropathy; MIDD: monoclonal immunoglobulin deposition disease; PGNMID: proliferative glomerulonephritis with monoclonal immunoglobulin deposition, AG: albumin globulin

Variables	All cases (n=41)	LCCN (n=19)	Amyloidosis (n=11)	MIDD (n=6)	PGNMID (n=5)	P value
Initial diagnosis of paraproteinemia by renal biopsy	32(78)	12(63)	11(100)	4(67)	5(100)	0.06^#^
Serum creatinine mg/dl	6.6±4.9	9.19±5.4	2.92±2.3	6.3±2.7	5.5±4.09	0.005*
24-hour urine protein gm/day	1.7 ±1.7	1.0 ± 0.6	3.0 ±2.6	1.6±1.4	1.3 ±1.1	0.01*
Nephrotic syndrome	7(17)	-	5(46)	1(17)	1(20)	0.04^#^
Serum AG ratio (mean ± SD)	0.97±0.4	0.8±0.5	1.0±0.24	1.14±0.4	0.96±0.08	0.4*
Tubular atrophy/interstitial fibrosis (mean ± SD)	1.2±0.7	1.21±0.7	1.09±0.9	1.33±0.8	1.2±0.8	0.9*

**Figure 1:**
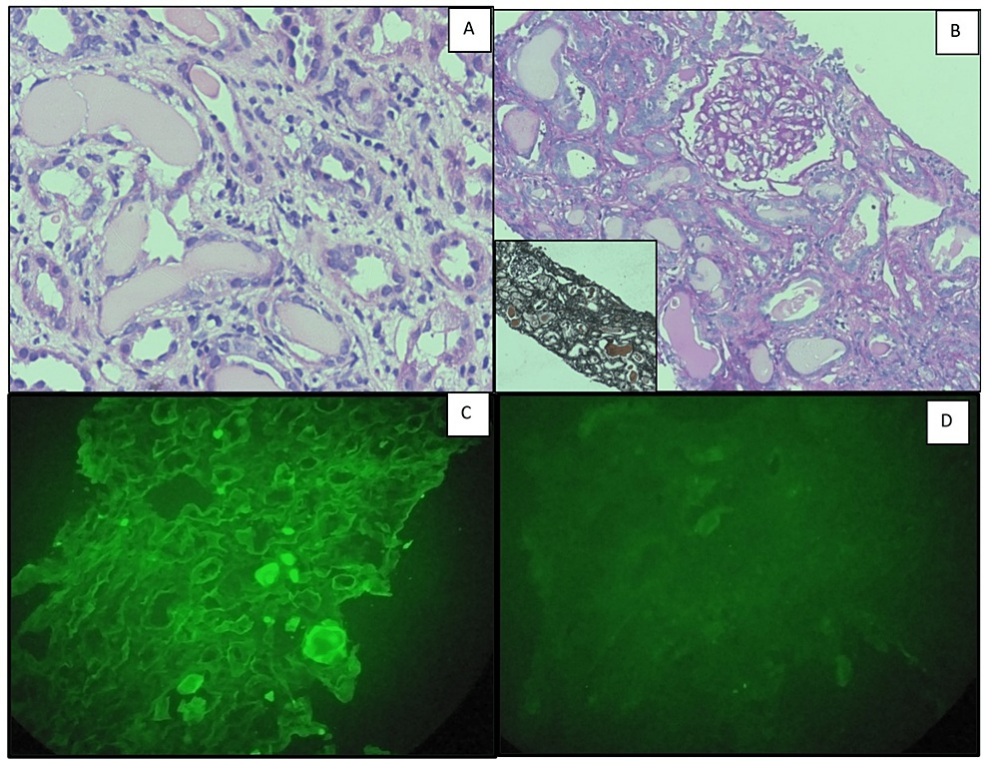
Photomicrographs of a case of light chain cast nephropathy. A: Fractured casts in kidney biopsy with mononuclear cell reaction (H&E X 400). B: PAS negative fractured casts in kidney biopsy(PAS X 200). Insert shows silver negative fractured casts (PASMX100). C: Kidney biopsy with fractured casts with kappa positivity (IF X 400) D: Kidney biopsy with lambda negativity (IF X 400) H&E: hematoxylin and Eosin; PAS: periodic acid-Schiff stain; PASM: periodic Schiff methenamine stain; IF: immunofluorescence.

Around 78% of the patients had an initial diagnosis suggesting monoclonal gammopathy on kidney biopsy (Table 2). The morphology of kidney biopsy in LCCN had pathognomonic PAS and silver-negative fractured casts with mononuclear cell reaction and monoclonal light chain restriction (Figure 1). GBM thickening and nodular mesangial expansion were seen in three (16%) overlapping cases in which two were LCCN + MIDD and the other with LCCN+ amyloidosis.

**Figure 2:**
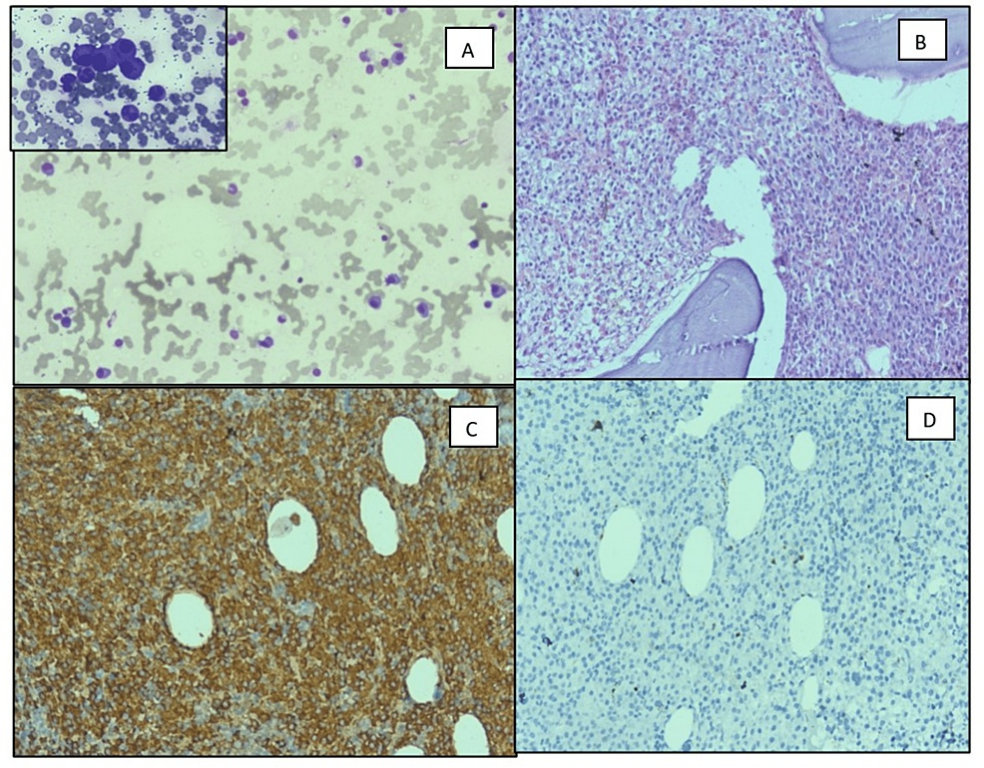
Bone marrow findings in a case with myeloma A: Bone marrow aspirate with increased plasma cells (BMA geimsa X 100, inset shows plasma cells in 400 X) B: Diffuse infiltration of marrow by plasma cells (BMB H&E X 200) C: Plasma cells with strong kappa positivity (IHC BMB Kappa X 200) D: Plasma cells with lambda negativity (IHC BMB Lambda X 200) BMA: bone marrow aspirate, BMB: bone marrow biopsy, IHC: immunohistochemistry

Bone marrow biopsy was available in 37 cases, of which myeloma (Figure 2) was concordant in 28 cases and amyloid in one while it was normal in eight cases. All patients of MIDD, LCCN, and 50% of amyloidosis cases had myeloma and all cases of PGNMID had normal marrow with a mean of 5% plasma cells.

Patients were followed for the maximum possible period/ till the end of the study period and the data is tabulated in (Table 3). These patients had received treatment according to the current treatment guidelines. Follow-up serum creatinine was taken as a measure for improving renal outcomes, the mean of which was 3.4mg/dl with a median follow-up of 12.4 months.

**Table 3:** Renal outcomes with follow-up (n=24) LCCN: light chain cast nephropathy; MIDD: monoclonal immunoglobulin deposition disease; PGNMID: proliferative glomerulonephritis with monoclonal immunoglobulin deposition

Variable	Findings	Total cases	LCCN	Amyloidosis	MIDD	PGNMID
Follow-up period in months Median±IQR		7±15.7 (n=24)	6±15 (n=12)	5.5±4 (n=6)	5±20 (n=5)	24 (n=1)
Follow-up creatinine in mg/dl Mean±SD		3.4±2.7 (n=41)	3.9±3.4 (n=15)	2.0±1.4 (n=9)	4.3±1.9 (n=6)	1.5 (n=5)

Monoclonal gammopathy of renal significance (MGRS)

Five patients each with amyloidosis and PGNMID were qualified for MGRS with no detectable plasma cell clone except for light chain restriction in kidney biopsy. These cases had mean plasma cells of 6% in the bone marrow, no significant plasma cell clones, or serum paraprotein (Tables 4-5).

**Table 4:** Pathological, hematological and treatment variables in MGRS n refers to the total number of cases with the available details of the particular variable among the cases with MGRS. MGRS: monoclonal gammopathy of renal significance; AKI: acute kidney injury; RPRF: rapidly progressive renal failure; ERS: erythrocyte sedimentation rate; PGNMID: proliferative glomerulonephritis with monoclonal immunoglobulin deposition

Pathological, hematological, and treatment variables among MGRS cases	Observed values
Kidney biopsy (n=10)	
Amyloidosis (AL type)	5(50%)
PGNMID	5(50%)
Provisional clinical diagnosis (n=10)	
AKI	2(20%)
RPRF	3(30%)
Nephrotic syndrome	1(10%)
Sub-nephrotic proteinuria	4(40%)
Mean Hb (n=10) (Mean±SD)	9.3±1.6mg/dL
Mean ESR (n=4) (Mean±SD)	77.8±26.9mm
Bone marrow findings (n=9)	
Normal marrow	8(88%)
Amyloidosis	1(12%)
Mean % of plasma cells in marrow (n=9) (Mean±SD)	6.3±3.2
The pattern of plasma cells infiltration in the marrow (n=9)	
Interstitial	9(100%)
Rouleaux formation (n=10)	0(0%)
Treatment received (n=5)	
Chemotherapy	3(60%)
Supportive therapy	2(40%)

**Table 5:** Demographic and biochemical variables in MGRS n is the total number of cases with available details of the particular variable among the cases with MGRS MGRS: monoclonal gammopathy of renal significance; AG albumin globulin; SPEP: serum protein electrophoresis; IFE: immunofixation electrophoresis

Demographic and biochemical variables	Observed values
Mean age (n=10) (mean ± SD)	60.5±9.5 years
M:F ratio (n=10)	2.3:1
Mean serum creatinine (n=10) (mean ± SD)	3.8±3.3mg/dL
Mean serum calcium(n=10) (mean ± SD)	8.3±0.3mg/dL
Mean AG ratio (n=10) (mean ± SD)	0.98±0.14
24-hour urine protein (n=10) (mean ± SD)	2.1±1.7gm/day
SPEP (n=5)	negative (100%)
IFE-(n=4)	
κ	1(25%)
IgG, κ	1(25%)
IgG, λ	1(25%)
IgM, λ	1(25%)
Urine sediments (n=10)	0(0%)
Outcome variables	
Follow up months (n=4) (median±SD)	10.3±9.3months
Follow up creatinine (n=4) (mean ± SD)	2.3±1.3mg/dL

## Discussion

In our study, we observed a spectrum of paraprotein-associated kidney disease including LCCN (46%), amyloidosis (27%), MIDD (15%), and PGNMID (12%). Our study had a relatively higher incidence of PGNMID (12%) when compared to a study by Nasr et al [5].

Around 78% of the patients had an initial diagnosis suggesting monoclonal gammopathy on kidney biopsy which had no clinical suspicion. This was 100% in the cases with amyloidosis which could be due to the fact that 90% of amyloidosis patients were having renal impairments at the time of diagnosis, necessitating a kidney biopsy initially; this also was observed in a study by Gerth et al. [9]. Next was PGNMID with 80% of cases initially diagnosed by kidney biopsy due to the lone renal manifestations in these cases [5].

An overall male-to-female ratio was 3.1:1 and this male predominance was seen among all the disease types. Even though male predominance was common among kidney diseases with plasma cell dyscrasias, a slight female predominance was observed among the patients with amyloidosis by Montseny et al [10]. Studies on PGNMID observed equal proportions of males and females, but we observed a male predominance with a 4:1 ratio like Nasr et al [5].

The mean kidney sizes on ultrasonography among the different disease types were within normal limits in our cases. Kidney in the plasma cell dyscrasias can be either normal, increased, or decreased in size depending on pathology as well as the duration of the disease as per Jennette et al. [6] but, an autopsy series by Herrera et al. [11] in contrary to our study had described grossly enlarged kidneys in the cases of amyloidosis and small kidneys in cases of MCN and MIDD.

Biochemical findings

Serum calcium levels were within the normal range in all types but it is relatively higher in LCCN with a mean of 9.2 mg/dl which was similarly observed by Montseny et al. [10] and Gerth et al [9]. The AG ratio was lowest in LCCN in this regard studies have mentioned only serum albumin which was lowest in amyloidosis [3,9,10].

In amyloidosis six cases had shown monoclonal protein in immunofixation electrophoresis (IFE) among which only three cases had shown positive M-spike in serum protein electrophoresis (SPEP) which was due to the higher sensitivity of IFE in detecting monoclonal protein. However, only SPEP had shown a statistically significant difference for M-spike positivity among the various paraproteinemic disease types which was comparable to Gerth et al. [9].

Hematological findings

The predominant marrow diagnosis observed was myeloma constituting 76% overall; 100% of the LCCN and MIDD had myeloma; contrary to the observations of Lin et al [12]. Around 50% of the patients with amyloidosis had associated myeloma which is 20% less when compared to the findings by Fielder et al [13]. In our study, we have observed a consistent pattern of infiltration of plasma cells in bone marrow among the different subtypes of kidney disease. High ESR was seen in MIDD followed by LCCN, amyloidosis, and PGNMID; this is contrary to the observations of Nasr et al. [3] where it was highest in LCCN followed by MIDD and amyloidosis.

Pathological findings

**Figure 3:**
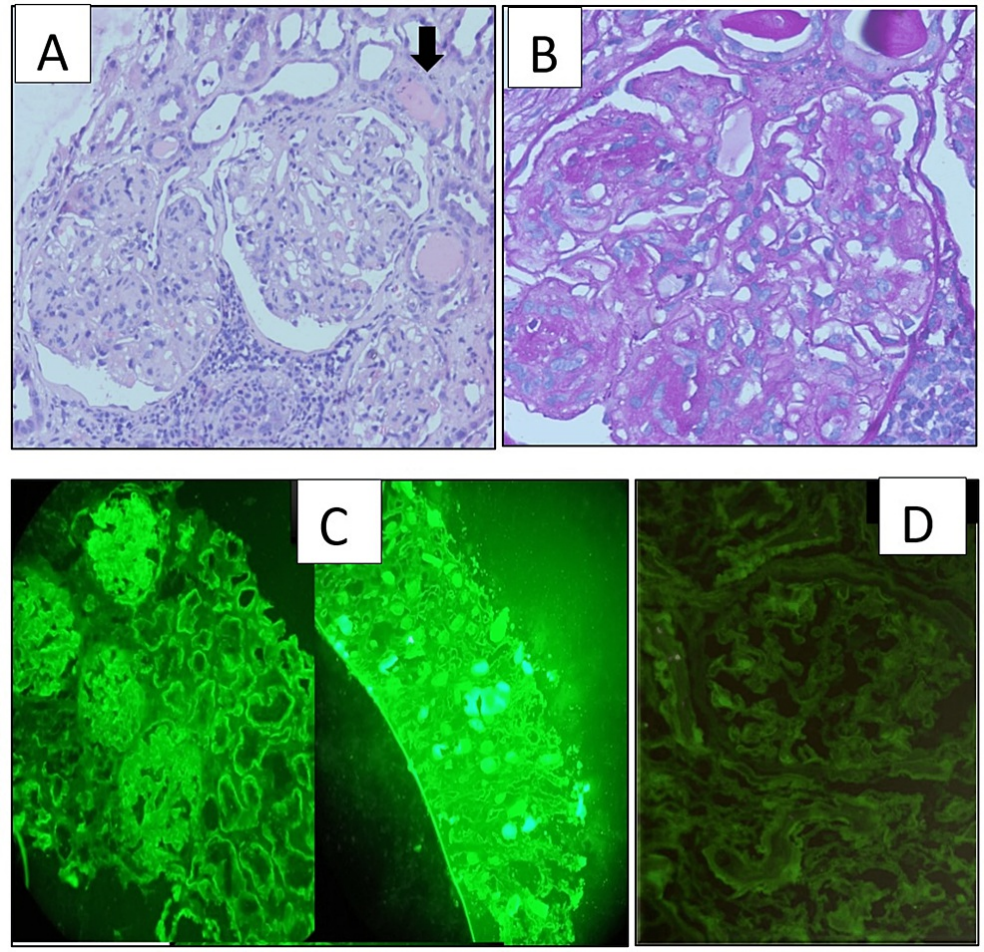
Photomicrographs of a case with mixed lesions - LCCN with MIDD A: Glomerulus with GBM thickening along with the light chain casts as shown by the arrow (H&E X 400) B: PAS positive with GBM thickening (PAS X 400) C: Kidney biopsy with Kappa positivity (The left image of C shows glomeruli with kappa positivity and right image shows kappa positive fractured casts) (IF Kappa X 20) D: Kidney biopsy with lambda negativity (IF lambda X 20) LCCN: light chain cast nephropathy; MIDD: monoclonal immunoglobulin deposition disease; GBM: glomerular basement membrane; H&E: hematoxylin and eosin; PAS: Periodic acid Schiff stain: IF: immunofluorescence

A total of 19 cases of light chain cast nephropathy were observed in our study out of which 16 cases had pure LCCN but three cases had mixed lesions; two with MIDD and one with amyloidosis. These mixed renal lesions in plasma cell dyscrasias are known and similar findings were observed by Lin et al. [12]. All three cases with mixed lesions had GBM thickening and mesangial nodules (Figure 3), this is in contrast to the observations by Lin et al. [12] where only 18% of cases with mixed lesions in MIDD had nodular sclerosing glomerulopathy, which might be due to their earlier presentation. On immunofluorescence, there was either κ (42%)or λ (54%) restriction, which was similar to Ecotiere et al [14].

**Figure 4:**
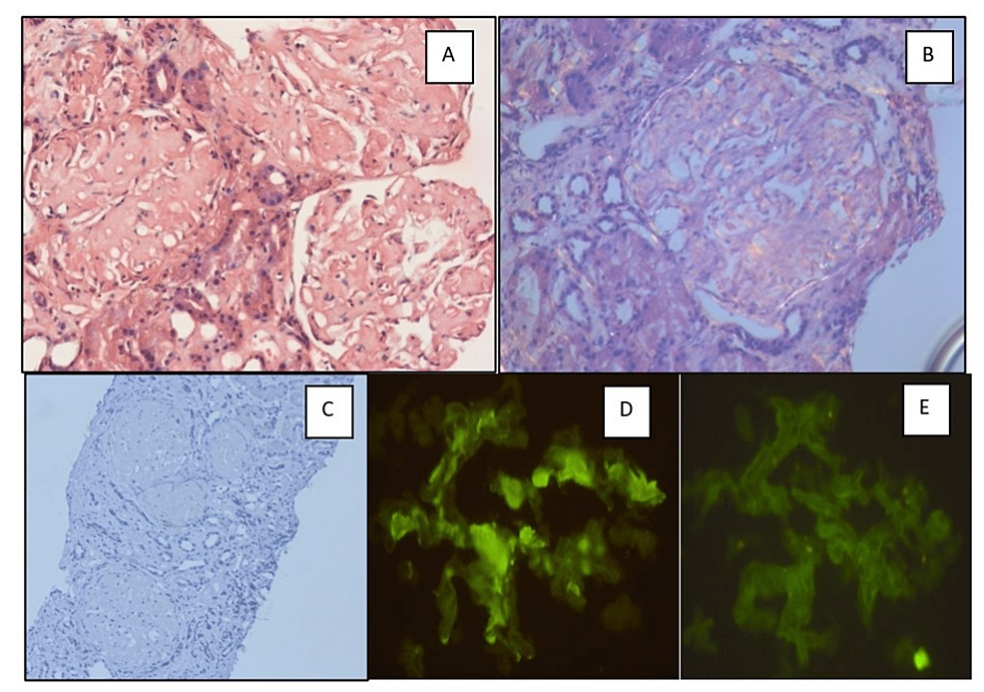
Photomicrographs of a case with amyloid light-chain (AL) amyloidosis A: Kidney biopsy with CR-positive amyloid deposits (CR X 200) B: Kidney biopsy with apple green birefringence of the amyloid  deposits (Polarizer X 200) C: Kidney biopsy with negative AA stain (IHC AA X 100) D: Kidney biopsy with lambda positivity (Lambda X 400) E: Kidney biopsy with kappa negativity (IF Kappa X 400) CR: Congo Red, IHC: immunohistochemistry, AA: amyloid A: IF: immunofluorescence

Morphological findings of amyloidosis(figure 4) are similar to Said et al., [15].On immunofluorescence, there is λ-light chain restriction in 66% which was similar to Gerth et al. [9].

**Figure 5:**
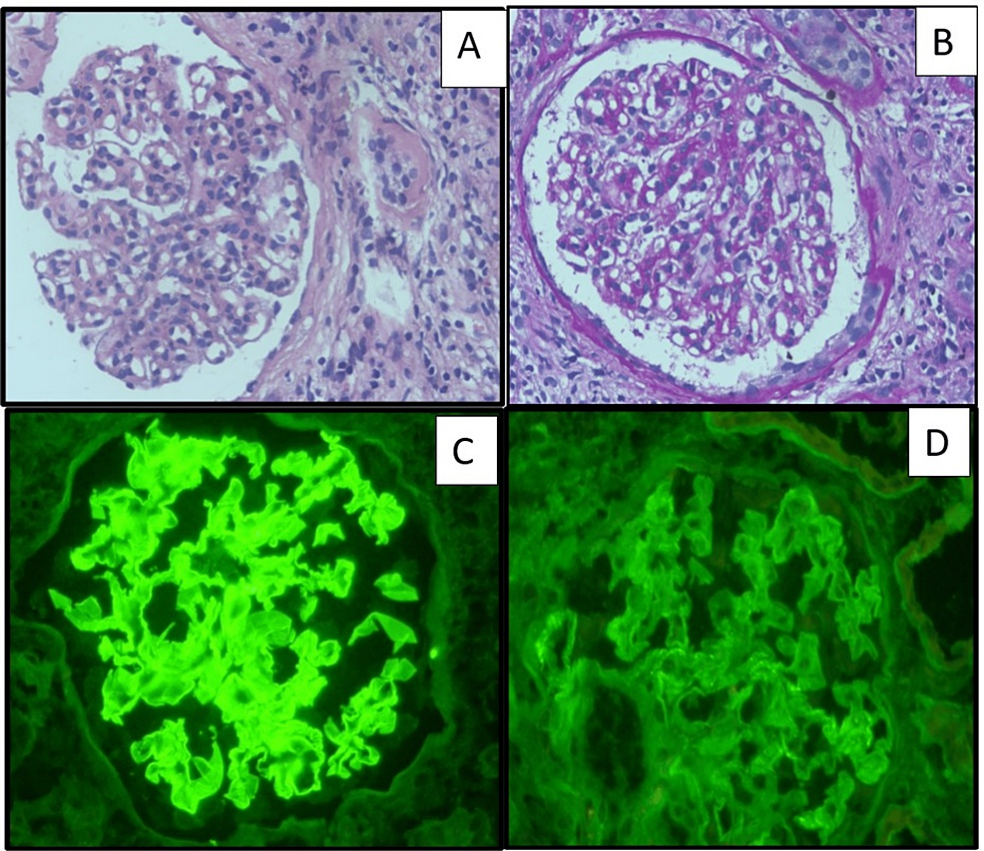
Photomicrographs of a case of monoclonal immunoglobulin deposition disease (MIDD) A: Kidney biopsy with GBM thickening and mild mesangial proliferation (H&E X 400) B: PAS-positive with GBM thickening (PAS X 400) C: Lambda positivity in GBM (IF Lambda X 400) D: Kappa negativity in GBM (IF kappa X 400) H&E: hematoxylin and eosin; PAS: periodic acid Schiff stain: GBM: glomerular basement membrane; IF: immunofluorescence

With regards to MIDD, the majority of the cases had shown GBM thickening and PAS-positive mesangial expansion (Figure 5), and three of them also showed nodules, in contrast to vascular changes in 83% of cases, similar to Lin et al. [12] where the predominant glomerular finding in MIDD is diffuse nodular mesangial expansion. Immunofluorescence had shown λ restriction in 50% of the cases which is contrary to the observations of Lin et al. [12] where 91% of cases with MIDD had κ- restriction.

**Figure 6:**
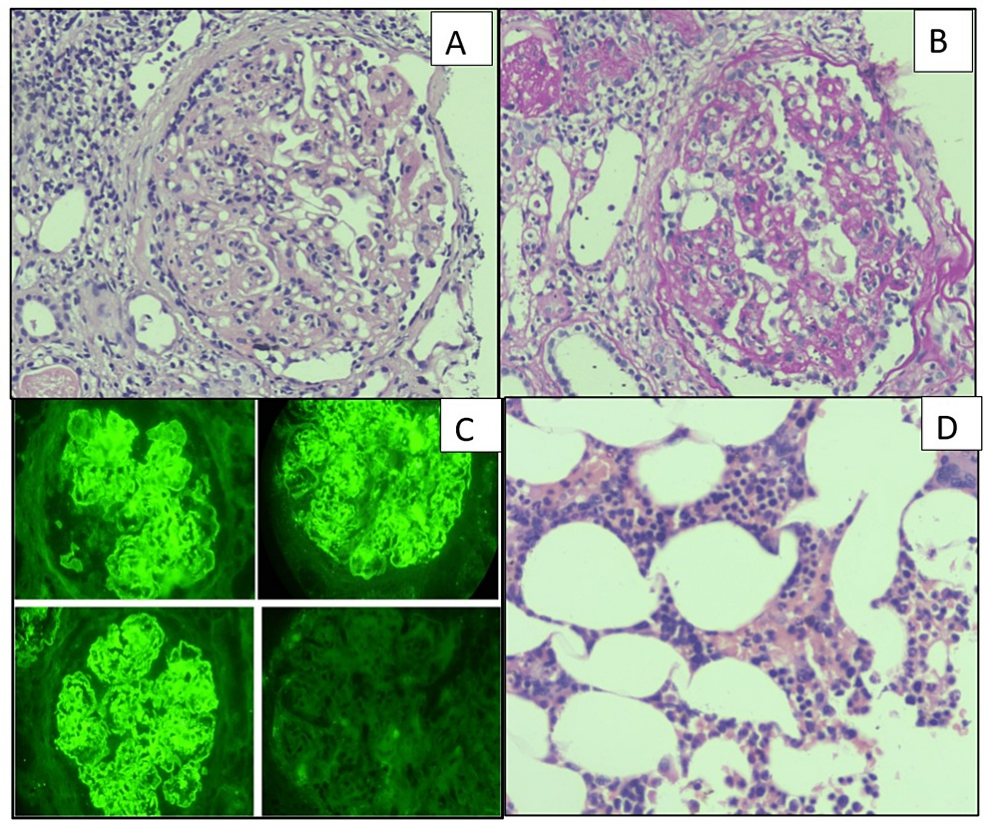
Photomicrographs of a case with proliferative glomerulonephritis with monoclonal immunoglobulin deposition (PGNMID) A: Kidney biopsy with endocapillary proliferation and irregular basement membrane thickening (H&E X 200) B: Glomerular changes highlighted by PAS (PAS X 200) C: IgG (top left), C3 (top right), and Lambda positivity (left bottom) with kappa negativity (bottom right) on IF (IF x 400). D: Bone marrow biopsy with only a slight increase in plasma cells (BMB H&E X 400). H&E: hematoxylin and eosin; PAS: periodic acid Schiff stain; IF: immunofluorescence; BMB: bone marrow biopsy

Diffuse endocapillary proliferative glomerulonephritis pattern was seen in all five cases with PGNMID (Figure 6). Also, two of them had shown irregular GBM thickening and mesangial proliferation. Vascular changes are not prominent with only mild to moderate fibrosis in 40% of cases which was similar to Nasr et al. [5]. Immunofluorescence had shown IgG, κ restriction in three cases and two cases had one each with IgM, λ, and IgA, λ restriction; however, only IgG with κ or λ restriction was observed by Nasr et al [5].

Monoclonal gammopathy of renal significance

We observed 10 patients satisfying the criteria for MGRS. These patients had mean plasma cells of 6% in the bone marrow and no significant serum paraprotein levels which is similar to other studies in MGRS [16-18]. Morphological findings in the kidney biopsy are according to the type of disease observed. Morphological types of kidney diseases observed in MGRS are more varied in Bridoux et al study. [7] in contrary to our study which could be attributable to a large number of cases in their study.

## Conclusions

Kidney biopsy is essential to establish the specific diagnosis, therapeutic, and important prognostic information in suspected cases of monoclonal gammopathy. The majority of the paraprotein-associated kidney disease cases in this study were initially diagnosed by kidney biopsy which signifies its importance. Paraprotein-associated lesions mainly LCCN, amyloidosis, and MIDD were encountered in kidney biopsies in a majority of these patients. These lesions had consistent findings among clinical and hematological variables. Even though hematological and biochemical investigations had detected monoclonal paraproteins in circulation, a subset of cases with low plasma cell burden and undetectable paraprotein in the serum but with monoclonal immunoglobulin deposition in the kidney like MGRS cases showed a reduction in the serum creatinine post-therapy.
